# Inhibition of BET bromodomain-dependent *XIAP* and *FLIP* expression sensitizes *KRAS*-mutated NSCLC to pro-apoptotic agents

**DOI:** 10.1038/cddis.2016.271

**Published:** 2016-09-08

**Authors:** Olaf Klingbeil, Ralf Lesche, Kathy A Gelato, Bernard Haendler, Pascale Lejeune

**Affiliations:** 1 Drug Discovery, Bayer Pharma AG, Berlin, Germany; 2 Institut für Biologie, Humboldt-Universität zu Berlin, Berlin, Germany

## Abstract

Non-small cell lung cancer (NSCLC) has the highest incidence of cancer-related death worldwide and a high medical need for more effective therapies. Small-molecule inhibitors of the bromodomain and extra terminal domain (BET) family such as JQ1, I-BET762 and OTX-015 are active in a wide range of different cancer types, including lung cancer. Although their activity on oncogene expression such as c-Myc has been addressed in many studies, the effects of BET inhibition on the apoptotic pathway remain largely unknown. Here we evaluated the activity of BET bromodomain inhibitors on cell cycle distribution and on components of the apoptosis response. Using a panel of 12 *KRAS*-mutated NSCLC models, we found that cell lines responsive to BET inhibitors underwent apoptosis and reduced their S-phase population, concomitant with downregulation of c-Myc expression. Conversely, ectopic c-Myc overexpression rescued the anti-proliferative effect of JQ1. In the H1373 xenograft model, treatment with JQ1 significantly reduced tumor growth and downregulated the expression of c-Myc. The effects of BET inhibition on the expression of 370 genes involved in apoptosis were compared in sensitive and resistant cells and we found the expression of the two key apoptosis regulators *FLIP* and *XIAP* to be highly BET dependent. Consistent with this, combination treatment of JQ1 with the tumor necrosis factor-related apoptosis-inducing ligand (TRAIL) or the pro-apoptotic chemotherapeutic agent cisplatin enhanced induction of apoptosis in both BET inhibitor sensitive and resistant cells. Further we showed that combination of JQ1 with cisplatin led to significantly improved anti-tumor efficacy in A549 tumor-bearing mice. Altogether, these results show that the identification of BET-dependent genes provides guidance for the choice of drug combinations in cancer treatment. They also demonstrate that BET inhibition primes NSCLC cells for induction of apoptosis and that a combination with pro-apoptotic compounds represents a valuable strategy to overcome treatment resistance.

In the last years, epigenetic regulators have become attractive targets for therapy of complex diseases like cancer, in which genetic and epigenetic alterations have essential roles. Since the approval of the first-generation epigenetic therapies targeting DNA methyltransferases and histone deacetylases for the treatment of malignancies and lymphomas, the field has expanded to several other protein families such as bromodomain proteins, histone methyltransferases and histone demethylases.^1^ Understanding and defining the roles of these epigenetic regulators at the cellular level is an important part of pre-clinical drug development. Bromodomain and extra terminal domain (BET) family (BRD2, BRD3, BRD4 and BRDT) inhibitors block the interaction between members of the BET family and acetylated lysine residues of histone tails.^2^ BET inhibitors such as JQ1, I-BET762 or OTX015 are active in a wide range of different cancer types.^3, 4, 5, 6, 7, 8, 9^ Their ability to reduce BRD4 occupancy at gene promoters and enhancers results in impaired transcription of cell-type-specific oncogene drivers.^10^ Strong responses in hematological malignancies and solid tumor models have been mainly attributed to suppression of oncogenes such as c-Myc.^3, 4, 5, 7, 10, 11, 12, 13, 14, 15, 16^ However, responses unrelated to c-Myc downregulation have also been reported, implying that other mechanisms are also involved.^17^

Regarding solid tumors, results from lung cancer models that respond to BET inhibitors have been published recently.^9^ About 80% of lung cancers can be histologically classified as non-small cell lung cancers (NSCLC),^18^ of which 30% harbor an activating oncogenic mutation in the GTPase domain of the *KRAS* signaling protein.^19^ Co-occurring genetic alterations of *KRAS* and the Liver kinase B1 (*LKB1*) tumor suppressor gene that lead to a more aggressive type of lung cancer with lower levels of immune markers, define a major subgroup (18–32%) of *KRAS*-mutated NSCLC.^20, 21^ This double-mutant subgroup has been reported to be *de novo* resistant to BET inhibitor treatment,^11^ but studies evaluating strategies to overcome resistance have not yet been reported.

Chemotherapy, usually including cisplatin or carboplatin treatment, is used to treat advanced NSCLC, however, with limited success. Resistance mechanisms by which a tumor evades drug-dependent cell death have been attributed to alterations in the apoptosis pathway.^22^ One of the key anti-apoptotic regulators overexpressed in NSCLC is the cellular FLICE-like inhibitory protein (c-FLIP), which binds to pro-caspase 8 and thereby regulates its activation by FADD (Fas-associated death domain protein) bound death receptors such as Fas, DR4, DR5 and TNF-R1.^23, 24^ In addition, cytoplasmic overexpression of c-FLIP has been linked to poor overall survival in NSCLC patients.^25^ Because of the structural similarity with caspase 8, direct targeting of c-FLIP with small molecules is challenging. Another frequently overexpressed anti-apoptotic regulator is X-linked inhibitor of apoptosis (XIAP), a member of the inhibitor of apoptosis protein (IAP) family, which blocks the activity of caspase-3, -7 and -9. Second mitochondria-derived activator of caspases (SMAC) can be released from mitochondria to inhibit XIAP function. Molecules that mimic SMAC are already in clinical development as apoptosis inducers or drug sensitizers of DNA damage chemotherapy.^26^

Here we investigated the effects of BET bromodomain inhibition in *KRAS*-mutated NSCLC and identified the transcriptional changes that conferred sensitivity or led to resensitization using combination approaches with pro-apoptotic agents. The combination of JQ1 and cisplatin further enhanced the effects of BET inhibition in JQ1-sensitive cells, while promoting apoptosis and reducing tumor growth in cells that showed a poor response to JQ1 alone.

## Results

### BET inhibitors show differential anti-proliferative activity in a panel of NSCLC cell lines

**Figure 1:**
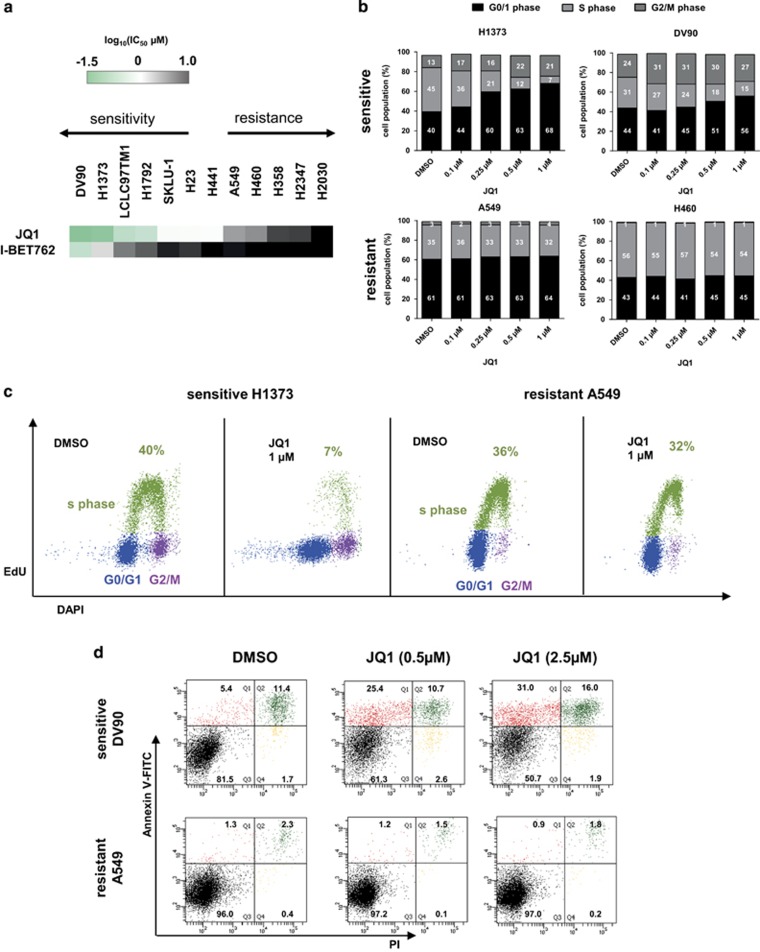
(**a**) IC_50_ values calculated from cell viability assays using *KRAS* mutant NSCLC cell lines after 72 h treatment with JQ1 or I-BET762. The data are represented as the mean IC_50_ of two or three independent experiments. (**b**) Cell cycle analysis of sensitive DV90 or H1373 and resistant A549 or H460 cells following 24 h of JQ1 treatment. Data are shown as the mean (*n*=2). (**c**) Representative cell cycle distribution of viable H1373 or A549 cells 24 h after 1 *μ*M JQ1 treatment. The S-phase cell population is shown in green, G0/G1 in blue and G2 in purple. (**d**) Flow cytometry results showing percentage of viable population and apoptotic cells of DV90 and A549 after 48 h JQ1 treatment using Annexin-V-FITC and PI staining. AV-positive only population (red), AV/PI-double-positive population in green, AV/PI-negative population in black and PI-positive only in yellow

To investigate the effects of the BET inhibitors JQ1 and I-BET762 on cell growth we treated a panel of 12 *KRAS*-mutated NSCLC cell lines for 72 h. On the basis of the half inhibitory concentration (IC_50_), we identified distinct subgroups of cell lines that were either particularly sensitive or resistant to BET inhibition (Figure 1a). To further characterize the effect of BET inhibitors on the cell cycle, a flow cytometry 5-ethynyl-2'-deoxyuridine (EdU) staining assay was performed in both sensitive and resistant cell lines. H1373 and DV90 cells showed a dose-dependent shift from S-phase towards G0/G1-population upon treatment (Figures 1b and c,Supplementary Figure. S1A), which was consistent with previous observations in lung adenocarcinoma and multiple myeloma cell lines.^9, 15^ Dose-dependent induction of apoptosis after JQ1 (Figure 1d,Supplementary Figure S1B) or OTX-015 (Supplementary Figure S1C) treatment was furthermore observed in sensitive DV90 and H1373 cells but not in resistant A549 and H460 cells irrespective of the p53 status of the cells. Interestingly, relative caspase 3/7 activity did not increase with longer exposure time from 24–72 h in H1373 cells (Supplementary Figure S1D). These results indicate that cell cycle arrest in sensitive cells is the primary early phenotype caused by BET inhibition, although induction of apoptosis takes place at a later stage.

### Differential regulation of the *MYC* oncogene by JQ1 in a panel of NSCLC cell lines

**Figure 2:**
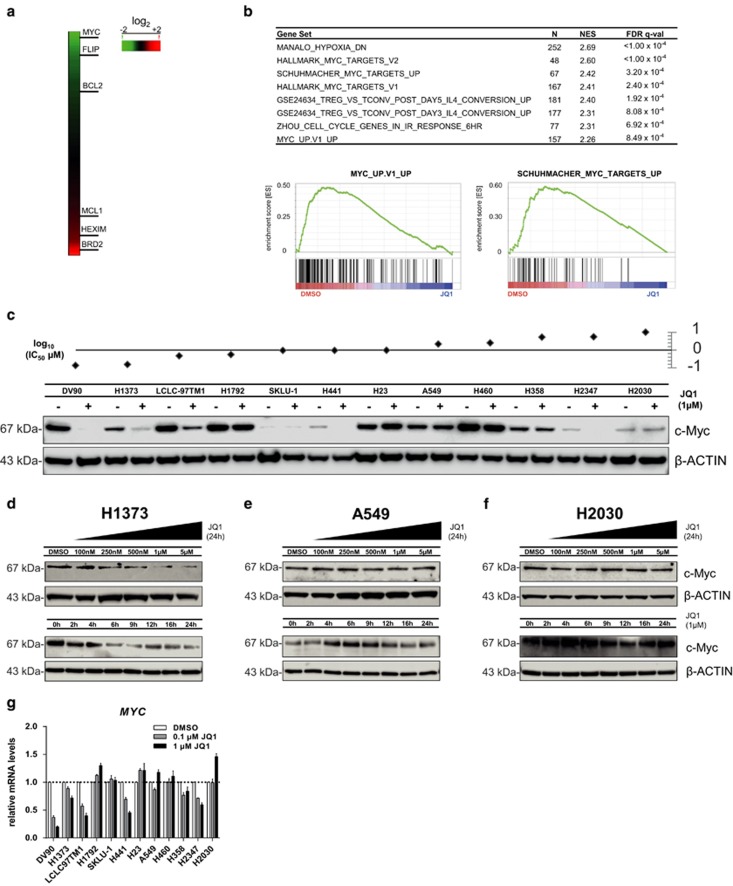
(**a**) Heat map of differentially expressed genes in DV90 cells 24 h after treatment with IC_50_ dose (135 nM) of JQ1. Microarray data were normalized and log fold difference compared to DMSO control is shown. (**b**) Gene set enrichment analysis (GSEA) of genes downregulated by JQ1 in DV90 cells. Top gene sets ranked by normalized enrichment score (NES), number of genes (*N*) and false discovery rate (FDR) are shown. Two representative *MYC* gene sets are depicted. (**c**) Western blot analysis of c-Myc level 24 h after DMSO (−) or 1 *μ*M JQ1 (+) treatment. Sensitivity of cell lines to JQ1 is represented by log (IC_50_ *μ*M). (**d–f**) Western blot analysis of c-Myc level in cell lines with different sensitivities to JQ1. H1373 (**d**) A549 (**e**) and H2030 (**f**) cells were treated with different doses of JQ1 for 24 h or 1 *μ*M JQ1 for different times. (**g**) Quantitative real time-PCR (qRT-PCR) analysis of *MYC* mRNA expression 24 h after treatment with 0.1 or 1 *μ*M of JQ1 normalized to housekeeping gene human cyclophilin A and DMSO-treated control. Error bars denote S.E.M. (*n*=3)

Using microarray profiling and gene set enrichment analysis (GSEA)^27^ of DV90 cells, we found that expression of the *MYC* oncogene and the anti-apoptotic *FLIP* and *BCL2* genes was downregulated (Figure 2a). We additionally identified the *MYC* transcriptional program to be highly represented among genes downregulated by BET inhibition (Figure 2b), confirming the results observed in earlier studies.^11^

We next characterized the protein levels of c-Myc in the NSCLC cell line panel 24 h after JQ1 treatment and found them to be reduced in the three most sensitive cell lines, although they were largely unaffected in resistant cell lines (Figure 2c). In the sensitive cell line H1373, time- and dose-dependent regulation of c-Myc by JQ1 was furthermore shown (Figure 2d), although c-Myc protein levels did not change in the resistant A549 and H2030 models (Figures 2e and f). We also confirmed downregulation of c-Myc at the messenger RNA (mRNA) level (Figure 2g), although neither basal *MYC* (supplementary Fig. S2B) nor expression levels of the BET family (Supplementary Figure S2C) were predictive of response to JQ1.

Consistent with previous observations^11^ resistant cell lines were frequently mutated in the tumor suppressor LKB1 (Supplementary Figure S2A). It is noteworthy that JQ1-dependent *FOSL1* downregulation, previously reported in lung cancer,^9^ was observed in both sensitive and resistant cell lines (Supplementary Figures S2D and S2E) and basal gene expression of *FOSL1* was also not predictive of response to JQ1 (Supplementary Figure S2B).

### Downregulation of c-Myc and anti-tumor efficacy of JQ1 are also observed *in vivo*

**Figure 3:**
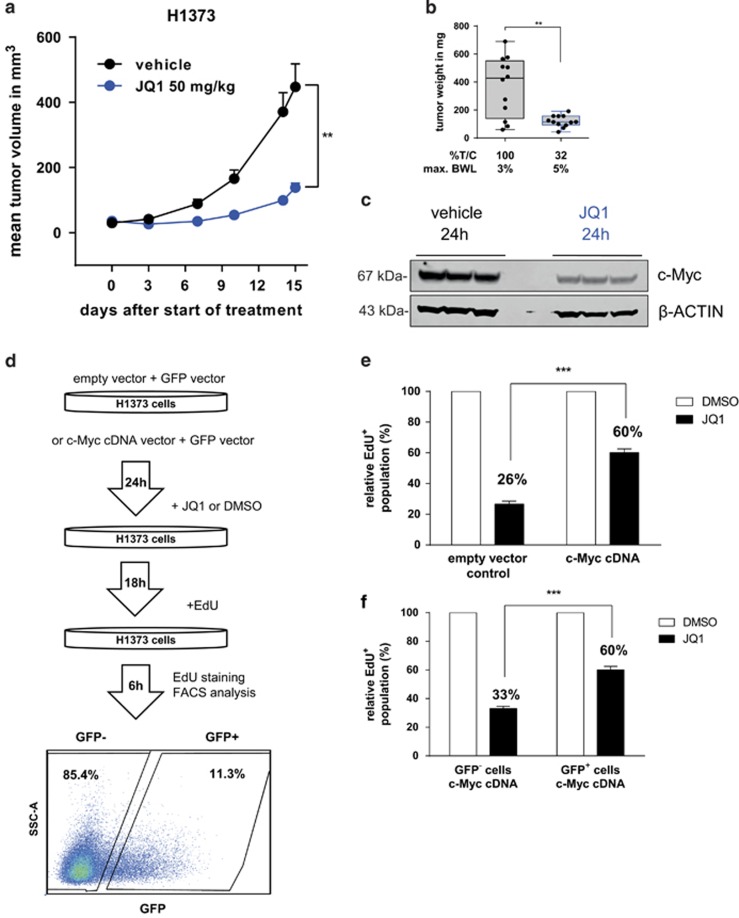
(**a**) Growth curve of H1373 xenograft treated with vehicle or 50 mg/kg JQ1 i.p. daily. Error bars denote S.E.M. (*n*=12 mice per group) **P<0.01 two-tailed unpaired Student's *t*-test on log transformed tumor volume on day 15 after start of treatment. (**b**) Box and whiskers plot of tumor weight in the H1373 xenograft study on day 15 after start of treatment. Error bars denote S.E.M. (*n*=12 mice per group). **P<0.01 two-tailed unpaired Student's *t*-test on log transformed tumor weight. (**c**) Western blot analysis c-Myc level in tumor tissue from the H1373 xenograft study treated with vehicle or 50 mg/kg JQ1. (**d**) Workflow of rescue experiment in H1373. Cells were transfected with empty vector or c-Myc expressing vector and GFP vector, and then subsequently treated with JQ1 (0.5 *μ*M) or DMSO and stained using EdU. (**e** and **f**) Results of overexpression rescue experiments comparing empty vector *versus* c-Myc vector transfected cells (**e**) and GFP^−^ *versus* GFP^+^ cells co-expressing c-Myc (**f**) EdU^+^ population was normalized to DMSO-treated control sample. Error bars denote S.E.M. (*n*=3). ***P<0.001 two-tailed unpaired Student's *t*-test

The effects of JQ1 treatment on c-Myc downregulation and inhibition of cell proliferation, observed *in vitro*, were also analyzed *in vivo* in mice bearing established H1373 tumors, treated daily with vehicle or JQ1 (intra peritoneal (i.p.)) for 15 days. We found that JQ1 given at 50 mg/kg was active with a percent treatment *versus* control tumor weight ratio (%T/C) of 32% on day 15 after start of treatment (Figures 3a and b). JQ1 was well tolerated with a maximum mean body weight loss (BWL) of 5%. Downregulation of c-Myc, as seen in NSCLC cell lines, was confirmed in tumor tissue lysates using western blot analysis, 24 h after JQ1 treatment with 50 mg/kg (Figure 3c).

### c-Myc overexpression rescues the effects of JQ1 *in vitro*

The JQ1-sensitive H1373 cell line was used to investigate whether cells could be rescued from the anti-proliferative effect of JQ1 with ectopic overexpression of c-Myc. Therefore, H1373 cells were co-transfected with c-Myc and GFP expression vectors, and transfected cells were identified by GFP expression (GFP^+^ cells). Ectopic overexpression of c-Myc significantly increased the EdU^+^ population of JQ1-treated H1373 cells (60% EdU^+^ with c-Myc vector *versus* 26% EdU^+^ with empty vector control; Figures 3d–f and Supplementary Figure S3). As ectopic expression of c-Myc could not completely rescue the effect of JQ1, we further analyzed whether BET inhibition additionally affected the apoptosis pathway.

### Expression of the apoptosis regulators c-FLIP and XIAP is dependent on BET proteins

**Figure 4:**
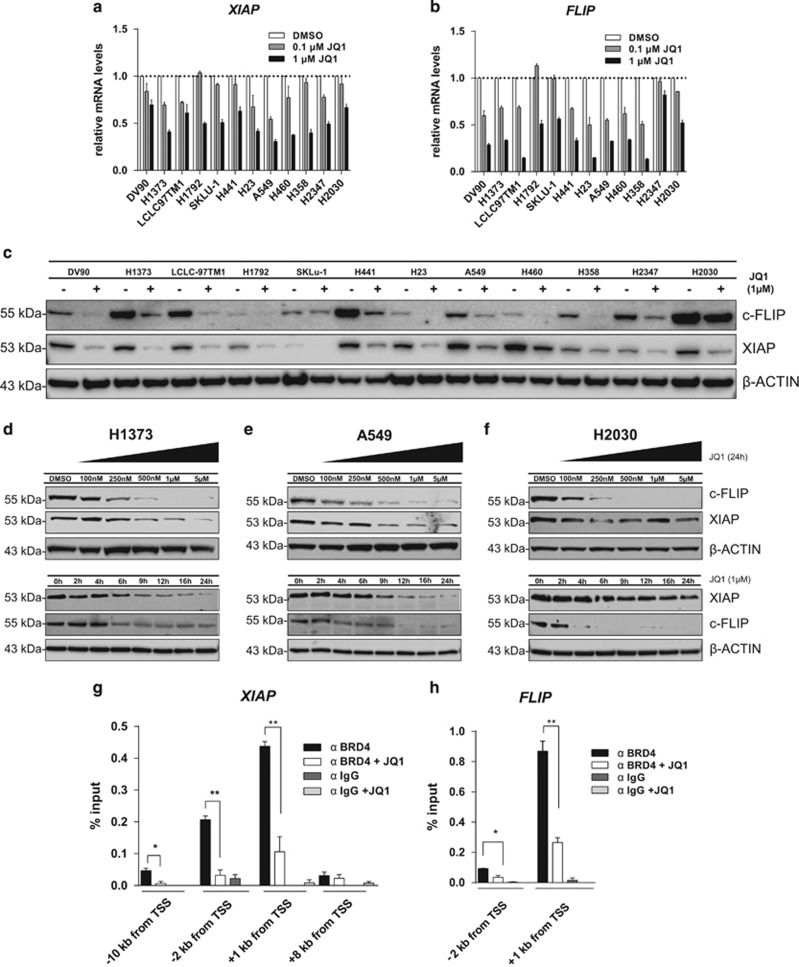
(**a** and **b**) Quantitative real time-PCR (qRT-PCR) analysis of *XIAP and FLIP* mRNA expression 24 h after treatment with 0.1 or 1 *μ*M of JQ1 normalized to both the expression levels of the housekeeping gene human cyclophilin A and DMSO-treated control. Error bars denote S.E.M. (*n*=3). (**c**) Western blot analysis of c-FLIP and XIAP 24 h after DMSO (−) or 1 *μ*M JQ1 (+) treatment. (**d**–**f**) Western blot analysis of c-FLIP and XIAP in cell lines with different sensitivities to JQ1. H1373 (**d**) A549 (**e**) and H2030 (**f**) cells were treated with increasing doses of JQ1 for 24 h or with 1 *μ*M for different times. (**g** and **h**) ChIP-qPCR analysis of BRD4 binding at the *XIAP* and FLIP promoters (primer distance from transcription start site (TSS) is indicated) in H1373 cells treated with DMSO or JQ1 (0.5 *μ*M). Error bars denote S.E.M. (*n*=3). *P<0.05, **P<0.01, two-tailed unpaired Student's *t*-test

We compared the transcriptional regulation of 370 apoptosis pathway genes in the JQ1-sensitive H1373 cells and the JQ1-resistant H2030 cell lines. Cells were treated with 1 *μ*M JQ1 for 6 h, followed by analysis of gene expression. We observed that the expression of two cellular caspase inhibitors *FLIP* and *XIAP*, which exhibit key regulatory functions in the apoptosis pathway, was strongly reduced in both cell lines (Supplementary Figure S4A). Interestingly, JQ1 reduced the mRNA levels of *FLIP* and *XIAP* in all NSCLC cell lines tested (Figures 4a and b). Reduction of both c-FLIP and XIAP at the protein level was also observed in all NSCLC cell lines tested after addition of 1 *μ*M JQ1 for 24 h (Figure 4c). Time-course and dose–response experiments confirmed the effect of BET inhibition on the protein levels in three cell lines with different sensitivities to JQ1 (Figures 4d–f). However, the expression of anti-apoptotic proteins such as members of the BCL2 and of the IAP family remained largely unaffected by BET inhibition in most of the cells tested (Supplementary Figures S4B–D).

We performed chromatin immunoprecipitation (ChIP) in H1373 cells and found that BRD4 occupancy at promoter sites of the genes coding for cellular caspase inhibitors *FLIP* and *XIAP* was significantly reduced by JQ1 (Figures 4g and h), in line with the reduction of expression. This suggests a link between BRD4 bromodomain inhibition dependent loss of *XIAP* and *FLIP* expression and increased caspase activity, as seen in H1373 cells (Supplementary Figure S1D). This increased susceptibility to caspase-dependent activation of apoptosis, which follows treatment with a BET inhibitor, may be linked to BIM upregulation (Supplementary Figure S4A),^28, 29^ but most likely needs additional triggers like c-Myc oncogene downregulation to effectively induce apoptotic cell death.

### BET inhibitor-dependent downregulation of XIAP and c-FLIP leads to enhanced TRAIL-induced apoptosis in all cell lines

**Figure 5:**
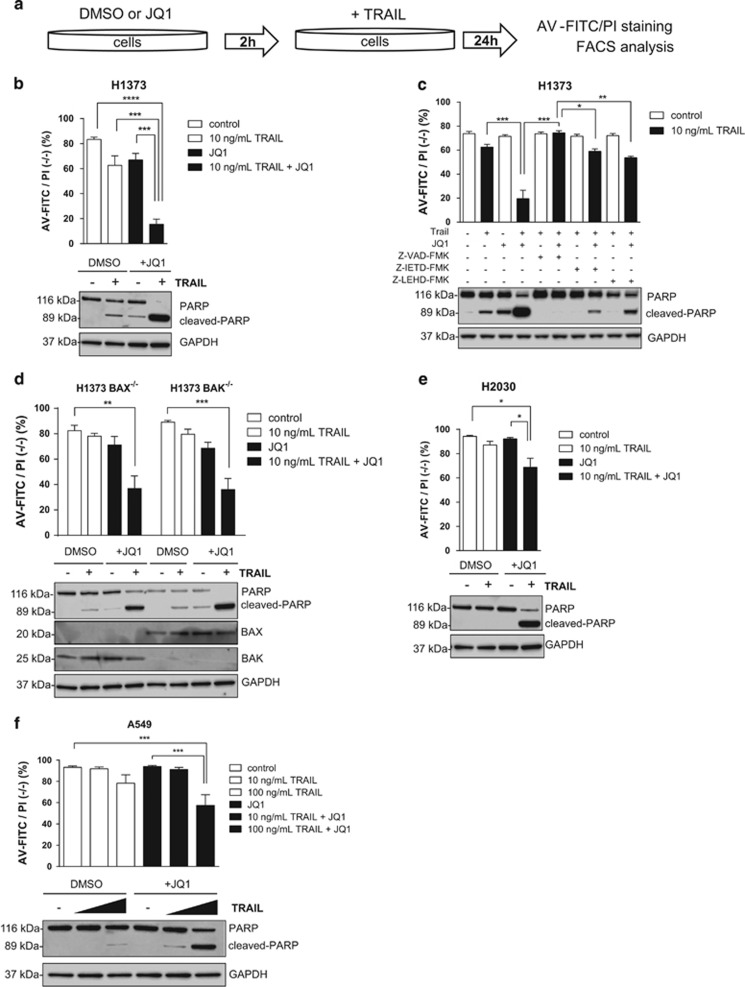
(**a**) Workflow of combination treatment using JQ1 and TRAIL. Cells were pre-treated with JQ1, subsequently treated with TRAIL, and stained using AV-FITC/PI for analysis of apoptosis with flow cytometer (results showing the percentage of viable population (%AV-FITC/PI negative (−/−)) are reported in top of (**b**–**f**); western blot results showing PARP cleavage are on the bottom of **b–f**). (**b**) Treatment of H1373 cells with TRAIL (10 ng/ml) and JQ1 (1 *μ*M) alone or in combination. (**c**) As in (**b**), however, with 20 *μ*M of the caspase inhibitors z-VAD-FMK (pan-caspase inhibitor), z-IETD-FMK (caspase 8 inhibitor) or z-LEHD-FMK (caspase 9 inhibitor). (**d**) Results of the rescue experiment after treatment of H1373 BAX or BAK knockout cells with TRAIL (10 ng/ml) and JQ1 (1 *μ*M) alone or in combination. (**e** and **f**), H2030 or A549 cells after treatment with TRAIL (10 or 100 ng/ml) and JQ1 (1 *μ*M) alone or in combination. Error bars denote S.E.M. (*n*=3–5). *P<0.05, **P<0.01, ***P<0.001, non-parametric one-way analysis of variance (ANOVA) with Sidak's correction for multiple comparisons

c-FLIP and XIAP exhibit key inhibitory functions in the extrinsic apoptosis pathway and were reduced in both resistant and sensitive cells treated with JQ1, although their loss did not consistently lead to apoptosis in all cell lines. We therefore combined JQ1 with death receptor ligand TRAIL (tumor necrosis factor-related apoptosis-inducing ligand) to test whether cell death can be stimulated, especially in the insensitive cell lines (Figure 5a). Death receptor binding of TRAIL is known to strongly activate the extrinsic apoptosis pathway by caspase 8/10 cleavage, leading to downstream activation of caspase 3 and amplifying activation of the intrinsic apoptosis pathway. Significantly enhanced TRAIL-dependent induction of apoptosis was evidenced by Annexin-V staining and PARP-cleavage in H1373 cells (Figure 5b; Supplementary Figure 5A), confirming results of an earlier study.^30^ We extended our analysis to determine which components of the apoptotic response were affected by JQ1, and established that enhanced TRAIL-induced apoptosis was dependent on caspase activity in these cells. Addition of the pan-caspase inhibitor carbobenzoxyvalyl-alanyl-aspartyl fluoromethyl ketone (z-VAD-FMK) completely rescued H1373 cells from induction of apoptosis, while the caspase 9 inhibitor carbobenzoxyleucyl-glutamyl-histidyl-aspartyl fluoromethyl ketone (z-LEHD-FMK) and the caspase 8 inhibitor carbobenzoxyisoleucyl-glutamyl-threonyl-aspartyl fluoromethyl ketone (z-IETD-FMK) could only partially rescue the effect (Figure 5c). Enhanced TRAIL-induced apoptosis was independent of pro-apoptotic proteins BAX or BAK, as knockout did not significantly affect the combinatorial potential of JQ1 and TRAIL (Figure 5d).

In order to confirm that the enhancing effect of JQ1 was linked to the downregulation of XIAP and c-FLIP, we performed small interfering RNA (siRNA) knockdown. Simultaneous knockdown of c-FLIP and XIAP was able to recapitulate the enhancing effects of JQ1 when combined with TRAIL in H1373 cells (Supplementary Figure S5B). JQ1 treatment also increased TRAIL-induced apoptosis in JQ1-insensitive cell lines H2030 and A459, and was able to overcome resistance (Figures 5e and f). To confirm this result, we treated five NSCLC models with different sensitivities to BET inhibition, either with JQ1 alone or in combination with TRAIL (Supplementary Figure S5F–J). Importantly, combination of TRAIL and JQ1 (Supplementary Figure S5E) did limit the viability of all five NSCLC models tested, whereas the combination did not reduce the viability of normal fibroblast cells (Supplementary Figure S5K).

### Combination of JQ1 and cisplatin synergistically reduces cell viability and overcomes resistance in the A549 xenograft

**Figure 6:**
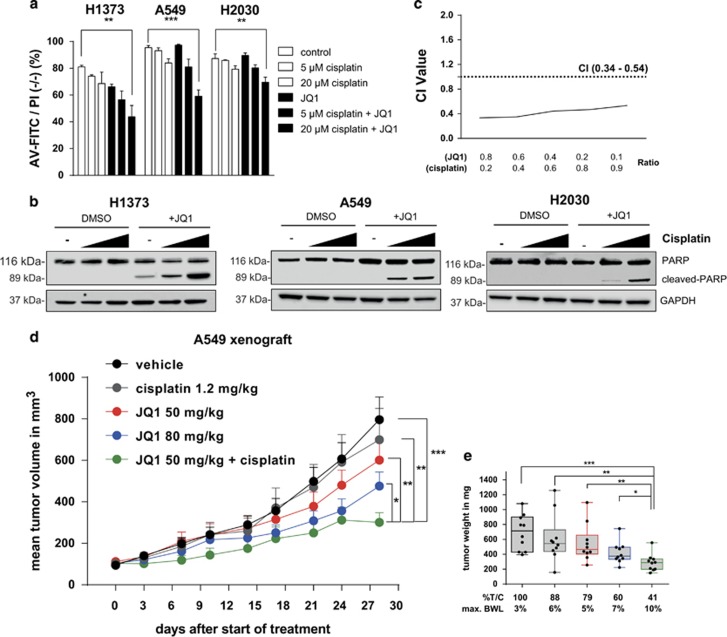
(**a**) Flow cytometry results showing the percentage of viable population (%AV-FITC/PI negative (−/−)) after treatment of H1373, A549 and H2030 cells with cisplatin and JQ1 (1 *μ*M) alone or in combination. Error bars denote S.E.M. (*n*=3). **P<0.01, ***P<0.001, non-parametric one-way analysis of variance (ANOVA) with Sidak's correction for multiple comparison. (**b**) Western blot analyses of PARP cleavage following treatment with cisplatin and JQ1 (1 *μ*M) alone or in combination. (**c**) Assessment of the degree of synergy between cisplatin and JQ1 in A549 cells using the Chou Talalay method. Calculated CI is plotted against drug ratios. Results are shown as the mean (*n*=2). The cutoff point for synergy is defined by CI<1.0. (**d**) Growth curve of A549 xenograft treated with vehicle, JQ1 (50 mg/kg, 80 mg/kg) i.p. daily, cisplatin 1.2 mg/kg i.v. QDx5 or in combination (50 mg/kg JQ1+1.2 mg/kg QDx5 cisplatin). Error bars denote S.E.M. (*n*=10 mice per group) *P<0.05, **P<0.01, ***P<0.001 two-tailed unpaired Student's *t*-test on log transformed tumor volume on day 28 after start of treatment. (**e**) Box and whiskers plot of tumor weight of the A549 xenograft study on day 28 after start of treatment. Error bars denote S.E.M. (*n*=10 mice per group). *P<0.05, **P<0.01, ***P<0.001 two-tailed unpaired Student's *t*-test on log transformed tumor weight

The sensitizing effect of BET inhibition towards pro-apoptotic agents through downregulation of c-FLIP and XIAP led us to explore the combination of JQ1 with pro-apoptotic chemotherapy such as cisplatin, which is frequently used as a single agent to treat NSCLC patients. Combination of JQ1 and cisplatin significantly increased apoptosis in all three cell lines tested, as measured by Annexin-V staining and PARP cleavage (Figures 6a and b; Supplementary Figure S6). Furthermore, cisplatin synergistically reduced viability of A549 cells when combined with JQ1, with a combination index (CI) of 0.34–0.54 (Figure 6c).

We then evaluated whether JQ1 together with cisplatin was more potent in reducing tumor growth compared with the single-agent treatment, by treating SCID mice bearing established A549 subcutaneous tumors with vehicle, cisplatin, JQ1, or JQ1 and cisplatin in combination (Figure 6d). Vehicle and JQ1 at 50 or 80 mg/kg (maximal tolerated dose) were given i.p. daily for 28 days and cisplatin was administered at its optimal schedule and dose at 1.2 mg/kg for 5 days (intravenous (i.v.)). The combination of JQ1 and cisplatin was given for 5 days within the first week of treatment, followed by JQ1 maintenance from day 14 until the end of the study on day 28. Cisplatin and JQ1 were well tolerated as single agents with a maximum mean BWL of 6% for cisplatin, and 5% and 7% for JQ1 at 50 and 80 mg/kg, respectively. Combination of cisplatin and JQ1 led to slightly increased mean BWL of 10%. The mice were therefore given 1-week drug holiday before starting the JQ1 maintenance. The activity of cisplatin and JQ1 alone in this study was classified as not active according to standard National Cancer Institute criteria with a %T/C of 88% for cisplatin, and 79% and 60% for JQ1 at 50 and 80 mg/kg, respectively (Figure 6d). However, the combination of cisplatin and JQ1 followed by JQ1 maintenance was active with a %T/C of 41% on day 28, and resulted in lower tumor weight compared with either agent alone, with statistical significance (Figure 6e).

## Discussion

Given the complexity of solid tumors, it is possible that epigenetic therapies will only unveil their full potential in the clinic when combined with other therapies. Recent pre-clinical studies evaluated combination partners of BET inhibitors such as phosphatidylinositol-4,5-bisphosphate 3-kinase inhibitors, histone deacetylase inhibitors, or a CD20 monoclonal antibody in different tumor types.^31, 32, 33^ Here we used the small-molecule BET inhibitor JQ1 alone and in combination with TRAIL or cisplatin in *KRAS*-mutated NSCLC cell lines, to promote apoptosis in these cells.

We characterized JQ1 in a panel of *KRAS* mutant NSCLC cell lines for its anti-tumor activity as well as its effect on c-Myc expression. Our results were in agreement with recent findings describing the efficacy of BET inhibitors in a *KRAS*-driven NSCLC mouse model.^11^ Using GSEA, we confirmed that the *MYC* transcriptional program was altered in JQ1-treated DV90 cells. Overexpression rescue experiments allowed us to link the strongly reduced proliferation of NSCLC cell lines induced by JQ1 treatment to c-Myc protein levels. On the basis of our findings we conclude that high sensitivity to JQ1 in *KRAS*-mutated NSCLC is mediated by concomitant high expression and JQ1-dependent downregulation of c-Myc. BRD4 was reported to occupy large enhancer sites (super enhancers) that define cell identity.^34, 35^ Loss of BRD4 can destabilize protein complexes that bind to these enhancers, which leads to strongly reduced expression of specific genes. In acute myeloid leukemia (AML) a distant super enhancer was identified that drives *MYC* expression in a BRD4-dependent manner.^10, 16^ The differential downregulation of *MYC* upon JQ1 treatment among the NSCLC models tested may be explained by the occurrence of different enhancer landscapes with variable dependencies on BRD4 occupancy.

Even though downregulation of the *MYC* oncogene can strongly reduce the growth of *KRAS* mutant NSCLC, we observed that only a distinct subgroup of NSCLC was affected in this way by JQ1 treatment. Constant expression of c-Myc in spite of treatment with BET inhibitors may have a role in inherent JQ1 resistance. In addition, recent findings suggest that BRD4-independent rebound of *MYC* expression is a mechanism of acquired resistance to BET inhibitor treatment in AML models with strong c-Myc dependency.^36^

Exploring the potential of BET inhibition beyond regulation of the *MYC* oncogene, we identified the reduced expression of the anti-apoptotic genes *FLIP* and *XIAP* as a common response to JQ1 treatment in NSCLC. In contrast to *MYC*, the expression of these two genes seems to be strongly BET protein-dependent. Death receptor targeting agents inducing extrinsic apoptosis are currently in clinical development^37^ and downregulation of two key anti-apoptotic proteins MCL1 and c-FLIP by CDK9 inhibition was recently identified to have enhanced effects in NSCLC, when combined with death-receptor ligand TRAIL.^38^ This result led us to evaluate the combination of JQ1 treatment with TRAIL. While finishing our studies another group also proposed that JQ1 enhances TRAIL-induced apoptosis,^30^ although the molecular mechanism remained largely unknown. We identified the simultaneous loss of the key apoptotic proteins XIAP and c-FLIP as the major driver of caspase-8 and -9-dependent TRAIL-induced cell death by JQ1. The pro-apoptotic activity of BET inhibitors was described to be dependent on BAX and BAK function.^39^Although BAX and BAK have similar but not completely redundant functions in induction of TRAIL-induced apoptosis,^40^ we observed that the effects of the combination of JQ1 and TRAIL were not affected by the loss of either BAX or BAK. This finding is in agreement with previous observations showing that XIAP downregulation enhances TRAIL activity in a BAX/BAK-independent manner.^41^

Interestingly, combination of JQ1 with the pro-apoptotic agent cisplatin had a synergistic effect on apoptosis as well as cell viability, independent of the sensitivity of the cell line to JQ1 as a single agent. Although the combination of JQ1 and cisplatin resulted in broad effects on transcriptional programs and signaling pathways, regulation of the anti-apoptotic protein c-FLIP was identified as a key driver for enhanced cell death induction. The increased anti-tumor effect also translated *in vivo* in the A549 xenograft model, while treatment with either cisplatin or JQ1 alone showed limited activity. Given the promising first results of the clinical phase I trials of BET inhibitors^42, 43, 44^ and that the biological diversity of solid tumors often leads to the ability to adapt to treatments, our results show that characterization of the effects of BET inhibition on genes involved on apoptosis can be valuable for the identification of attractive combination partners. In addition, our pre-clinical results provide evidence that epigenetic therapy targeting BET proteins, alone or in combination with novel or standard therapies, may be a useful approach to improve outcomes and overcome resistance compared with current standard-of-care treatments for NSCLC patients.

## Materials and Methods

### Cell lines and culture conditions

All cell lines used in this study were purchased from the American Type Culture Collection or the Deutsche Sammlung von Mikroorganismen und Zellkulturen GmbH (DMSZ, Braunschweig, Germany). The H441 cell line was from the Bayer cell stock and has been authenticated by finger printing at the DSMZ. Cells were maintained in RPMI 1640 (Biochrom, Berlin, Germany) or DMEM/F12 (Biochrom) supplemented with 10% (v/v) FCS (Biochrom). Non-essential amino acids (Biochrom) were added in the case of DV90. IMR-90 and Wi38 cells were cultured in MEM Earle's Medium with 10% fetal calf serum (FCS).

### Reagents and antibodies

Human MYC cDNA construct was purchased from OriGene (SC112715, pCMV6-XL5-MYC) and Tag-GFP construct from Evrogen (FP121, pTagGFP-C). Clustered regularly interspersed short palindromic repeats (CRISPR) plasmids were purchased from Santa Cruz Biotechnology: BAX (sc-400042, BAX-KO-GFP); BAX HDR (sc-400042-HDR, BAX-HDR-RFP-Puro); BAK1 (sc-400646, BAK1-KO-GFP); BAK1 HDR (sc-400646-HDR, BAK1-HDR-RFP-Puro).

Chemicals and agents were from the following sources: MG132 (M7449-1ML, Sigma Aldrich, St. Louis, MO, USA), 4',6-diamidin-2-phenylidol (DAPI) (40043, Biotinum, Fremont, CA, USA), iz-TRAIL (AG-40B-0069-5010, Adipogen, San Diego, CA, US), Cisplatin (P4394-100MG, Sigma Aldrich), Puromycin (sc-108071, Santa Cruz Biotechnology), z-VAD-FMK, (G7231, Promega, Sunnyvale, CA, USA), z-IETD-FMK, (51-69401U, BD Biosciences, San Jose, CA, USA), z-LEHD-FMK, (51-69411U, BD Biosciences), JQ1, I-BET762 and OTX-015 were synthesized in house.

Immunoblotting antibodies were purchased as follows: anti-c-Myc (ab32, Abcam, Cambridge, UK), anti-FOSL1 (#5281, Cell Signaling, Danvers, MA, USA), anti-c-FLIP (7F10, Enzo Life Sciences, Exeter, UK), anti-BCL-XL (#2764, Cell Signaling), anti-Mcl1 (#5453, Cell Signaling), anti-BCL2 (#2870, Cell Signaling), anti-c-IAP1 (#7065, Cell Signaling), anti-c-IAP2 (#3130, Cell Signaling), anti-Survivin (#2808, Cell Signaling), anti-XIAP (#2045, Cell Signaling) anti-BID (#2002, Cell Signaling), anti-BAX (#5023, Cell Signaling), anti-BAK1 (#12105, Cell Signaling), anti-PARP (#9542, Cell Signaling), anti-GAPDH (ab9485, Abcam), anti- β-ACTIN (ab8227, Abcam). All antibodies were used at 1:500–1:1000 dilution except GAPDH (1:2000) and β-ACTIN (1:4000).

Chromatin immunoprecipitation (ChIP) antibodies were anti-BRD4 (A301-985A, Bethyl Laboratories, Montgomery, TX, USA) and rabbit IgG (R2004-5X1MG, Sigma).

### Drug treatment and determination of cell viability

Cells were seeded at pre-optimized density (between 1000–2000 cells/well in 96-well plate format or 350 cells/well in 384-well plate format) on the day before treatment and cultured in presence of drug or vehicle for 72 h. On-plate positive control wells were treated with 1 *μ*M MG132, a proteasome inhibitor toxic to most cell lines. Values were normalized to 100%, which was equivalent to the average of the negative control DMSO and to 0%, which was equivalent to the average of the positive control MG132. Viability was assessed in duplicate or triplicate using CellTiter-Glo One Solution (CTG) assay (Promega) according to the manufacturer's instructions. IC_50_ values were calculated using non-linear regression model in GraphPad Prism 6 (La Jolla, CA, USA). Resulting IC_50_ values were expressed as an average of two or three independent experiments. Combined drug effects were calculated by the combination-index (CI) analysis. The CI values were calculated from a range of drug ratios (*R*) and the corresponding IC_50_ values using the Chou Talalay method.


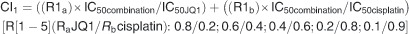


### Caspase 3/7 activity assay

Cells were seeded and treated as described above. After the indicated drug exposure time, caspase activity was assessed using Caspase-Glo 3/7 Assay (Promega) according to the manufacturer's instructions. Raw values were normalized to cell viability using CTG assay and fold change compared with DMSO control was calculated.

### Cell cycle analysis

Cells were seeded in six-well plates on the day before treatment and BET inhibitor or DMSO vehicle was added for the indicated time and dose. Cells were subjected to 10 *μ*M EdU 6 h before staining. Viable attached cells were then washed once with cold phosphate-buffered saline (PBS) and detached using Trypsin/EDTA solution (Biochrom), resuspended and filtered through a 70-*μ*m mesh (Corning, New York, NY, USA). The EdU^+^ population was determined using Click-iT EdU Alexa Fluor 647 Flow Cytometry Assay Kit (Life Technologies) and stained with DAPI at 1 *μ*g/ml final concentration for 1 h at 4 °C in the dark before flow cytometer analysis.

### Annexin V staining

Cells were treated and detached cells were pooled with attached cells as described above. Apoptosis was analyzed using the FITC Annexin-V Apoptosis Detection kit I (BD Biosciences CA) according to the manufacturer's instruction, followed by flow cytometer analysis.

### Western blot

Cells were seeded and treated as described above, washed in cold PBS, and then lysed using radioimmunoprecipitation assay (RIPA) buffer containing Halt Protease and Phosphatase inhibitor mixture (Thermo Fisher Scientific, Waltham, MA, USA) and 100 U/ml Benzonase. Lysates were incubated at 4 °C and then clarified by centrifugation at 13 000 × *g* for 10 min. The concentration was determined using Pierce BCA Protein Assay kit (Thermo Fisher Scientific). Equivalent amounts of lysate were analyzed by western blot.

### Quantitative reverse-transcriptase PCR

#### TaqMan

Cells were seeded and treated as described above. Total RNA was extracted using the RNeasy Plus Mini kit (Qiagen, Hilden, Germany) and cDNA was synthesized from 500–1000 ng total RNA using SuperScript III First-Strand Synthesis Supermix (Invitrogen, Carlsbad, CA, USA). TaqMan Gene Expression Assays (Life Technologies) were performed with the following primers: MYC (Hs00905030_m1); FLIP (Hs00153439_m1); XIAP (Hs00745222_s1); FOSL1 (Hs04187685_m1); BRD4 (Hs01006453_m1); BRD3 (Hs00201284_m1); BRD2 (Hs01121986_g1); human cyclophilin A (4326316E) using standard FastTaqMan Assay on the HT7900 system (Applied Biosystems, Foster City, CA, USA). Relative expression was calculated with the ΔΔCt method, using the average threshold for cyclophilin A to normalize gene expression between samples. All samples were measured in triplicate.

#### Apoptosis PCR array

Cells were seeded and treated as described above. Total RNA was extracted using the RNeasy Plus Mini kit and cDNA was synthesized from 500–1000 ng total RNA using RT^2^ First Strand Kit (Qiagen). Genes were analyzed on Human Apoptosis PCR Array 384HT (PAHS-30127) using SYBR Green ROX qPCR Mastermix (Qiagen) on the HT7900 system. Relative expression was calculated with the ΔΔCt method, using the average threshold for human B2M, GAPDH, HPRT1 and RPLP0 to normalize gene expression between samples.

### ChIP experiments

Cells were seeded in 300 cm^2^ culture flasks and treated with 500 nM JQ1 or DMSO vehicle for 6 h. Cells were washed and fixed in 1% formaldehyde for 10 min at room temperature before adding glycine. Cells were washed using cold PBS, collected, snap frozen and stored at −80 °C. Cells were lysed using lysis buffer containing Halt protease inhibitor. Chromatin was isolated using sonication buffer, sheared using a Covaris S220 device (10% duty cycle, intensity 5, cycle/burst 200) for 12 min at 20W and immunoprecipitated using 3 *μ*g of the indicated antibodies and bound to Dynabeads coated with protein A (Diagenode). Bound beads were washed three times with sonication buffer, one time with high-salt sonication buffer containing 500 mM NaCl, one time with LiCl-buffer, one time with TE-buffer. Bound complexes were eluted using elution buffer at 65 °C for 10 min. Crosslinking was reversed overnight at 65 °C followed by protein and RNA digestion using proteinase K and RNase A. DNA purification was done using QIAquick PCR Purification Kit (Qiagen). qPCR was performed using 2 × Maxima SYBR Green ROX qPCR Mastermix (#330521, Qiagen) on the HT7900 system as described above. ChIP-qPCR primers targeting human *XIAP* and *FLIP* promoter regions were purchased from EpiTect Qiagen: hXIAP NM_001167.2 at (−10 kb, −2 kb, +1 kb,+8 kb from transcription start site (TSS)), hFLIP NM_003879.4 at (−2 kb,+1 kb from TSS). Buffer composition used is shown in Supplementary Table 1.

### Gene expression profiling and GSEA

Cells were treated with DMSO or JQ1 and RNA was extracted as described above. Profiling was performed on Affymetrix HuGene-2.1ST arrays as previously described.^45^ Data are available at the GEO database (GSE75960). Briefly, probe set intensities were condensed to meta-probe set levels in Genedata Expressionist 9.0 using RMA algorithm followed by LOWESS normalization. To standardize, the median value for each gene across replicates was taken. GSEA (http://www.broadinstitute.org/gsea) was used to determine gene set enrichment of genes downregulated by JQ1 treatment in DV90 cells.^27^ To test which gene sets were associated with a given phenotype (here JQ1 treatment), all current gene sets from the Molecular Signatures Database were used (Msigdb.v5.0). Using a linear model, with treatment dose and duration as factors, statistical significantly regulated genes (Benjamini-Hochberg corrected *P* value (BH-q) < 10^-4^) for the effect of treatment were identified. Genes whose expression was dose-dependently altered were further normalized for differential expression (log_2_ fold change (FC) of expression from treatment *versus* vehicle samples). Significantly differentially expressed genes with a log_2_ FC cutoff of±0.5 are listed in Supplementary Table 2.

### Transfection of cell lines

For siRNA knockdown, cells were seeded on the day before transfection with ON-TARGETplus-SMARTpool siRNAs (GE Dharmacon, Lafayette, CO, USA) using Oligofectamine Transfection Reagent (Life Technologies), according to the manufacturer's instruction. The following SMARTpool-siRNAs were used (*n*=4): XIAP (L-004098-00-0010), c-FLIP (L-003772-00-0010), control siRNA (1027281, Qiagen). Plasmid DNA transfection was done as described above using Lipofectamine LTX with Plus reagent (Life Technologies).

### Gene knockout

Cells were co-transfected as described above using pooled CRISPR-Cas9 knockout (KO) plasmids (Santa Cruz Biotechnology) and homology-directed DNA repair (HDR) plasmids, corresponding to the cut sites generated by the CRISPR-Cas9 KO plasmids which coded for a puromycin resistance cassette and red fluorescent protein (RFP; Santa Cruz Biotechnology). Two days after transfection, stable knockout cells were selected using previously optimized dose of puromycin dihydrochloride (Santa Cruz Biotechnologies), and cultured for 2 weeks. Stable gene knockout was confirmed by western blot.

### Rescue experiments

H1373 cells were seeded 24 h prior to transfection. Cells were co-transfected as described above using a human c-Myc cDNA construct or empty control vector and TagGFP construct.^46^ Transfected cells were treated with 500 nM JQ1 for 24 h and stained using EdU-staining kit (Click-iT EdU Alexa Fluor 647 Flow Cytometry Assay Kit) as described above.

### *In vivo* mouse studies

All experiments were performed in accordance to the German Animal Welfare Law. Animals were housed following institutional guidelines. Six- to 8-week-old female SCID mice (Fox Chase CB17/Icr-Prkdc^scid^/IcrIcoCrl; Charles River) were acclimated for seven days before tumor cell injection. H1373 tumor cells were inoculated subcutaneously (s.c.) in female SCID mice at 3 × 10^6^ cells/mouse in 0.1 ml matrigel basement membrane matrix (BD biosciences)/medium (1:1). A549 tumor cells were inoculated s.c. in female SCID mice at 3 × 10^6^ cells/mouse in 0.1 ml matrigel. Tumor growth was assessed twice a week using a caliper and tumor volume was calculated using the formula: tumor volume=[length × width × width]/2. Tumor-bearing mice were randomized before start of treatment. Mouse body weight was determined at least twice a week before start of treatment and daily after start of treatment. Treated over control ratio (T/C) was calculated as mean tumor weight of treated group/mean tumor weight of vehicle control group at the end of the study (%T/C≤42% was declared active in agreement with National Cancer Institute criteria).^47^ For i.p. injection, JQ1 was dissolved in 30% 2-Hydroxypropyl-beta-cyclodextrin (HP-β-CD) in water, pH6 and applied daily with a volume of 10 m/kg.

### Statistical analysis

For statistical analysis of experiments, One-way analysis of variance with Sidak's correction for multiple comparisons or two-tailed *t*-test from GraphPad Prism 6.0 was used. Adjusted *P* values are indicated – *P<0.05, **P<0.01, ***P<0.001.

## Abbreviations

EdU: 5-ethynyl-2'-deoxyuridine
BET: Bromodomain and extra terminal domain
BRD2: Bromodomain containing protein 2
BRD3: Bromodomain containing protein 3
BRD4: Bromodomain containing protein 4
BRDT: Bromodomain containing protein in testis
z-IETD-FMK: carbobenzoxyisoleucyl-glutamyl-threonyl-aspartyl fluoromethyl ketone
z-LEHD-FMK: carbobenzoxyleucyl-glutamyl-histidyl-aspartyl fluoromethyl ketone
z-VAD-FMK: carbobenzoxyvalyl-alanyl-aspartyl fluoromethyl ketone
CTG: CellTiter-Glo
c-FLIP: cellular FLICE-like inhibitory protein
CRISPR: clustered regularly interspersed short palindromic repeats
DR4: Death receptor 4
DR5: Death receptor 5
DSMZ: Deutsche Sammlung von Mikroorganismen und Zellkulturen GmbH
FCS: fetal calf serum
GSEA: gene set enrichment analysis
IC_50_: half inhibitory concentration
i.p.: intraperitoneal
i.v.: intravenous
LKB1: Liver Kinase B1
mRNA: messenger RNA
NSCLC: non-small cell lung cancer
PBS: Phosphate-buffered saline
SMAC: Second mitochondria-derived activator of caspases
siRNA: small interfering RNA
TSS: transcription start site
TRAIL: tumor necrosis factor-related apoptosis-inducing ligand
KRAS: v-Ki-RAS Kirsten rat sarcoma viral oncogene
XIAP: X-linked inhibitor of apoptosis
