# Supplementary figures and images for: Inhibition of BET bromodomain-dependent XIAP and FLIP expression sensitizes KRAS-mutated NSCLC to pro-apoptotic agents
Source: Cell Death Dis. 2016 Sep 8;7(9):e2365–. doi: 10.1038/cddis.2016.271 (PMC5059870; doi:10.1038/cddis.2016.271)

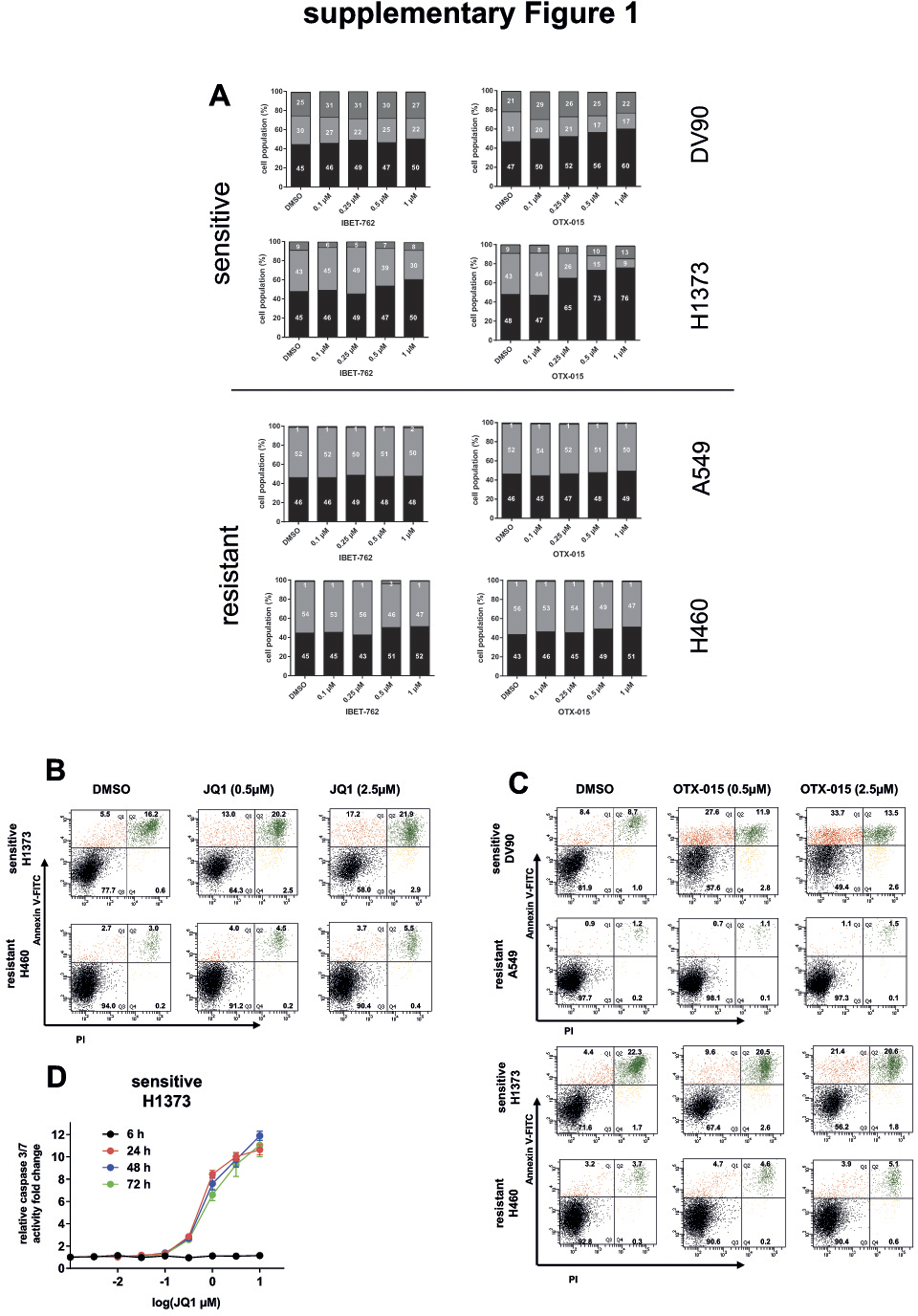

Supplement: Supplementary Figure 1 [file cddis2016271x1.tif]

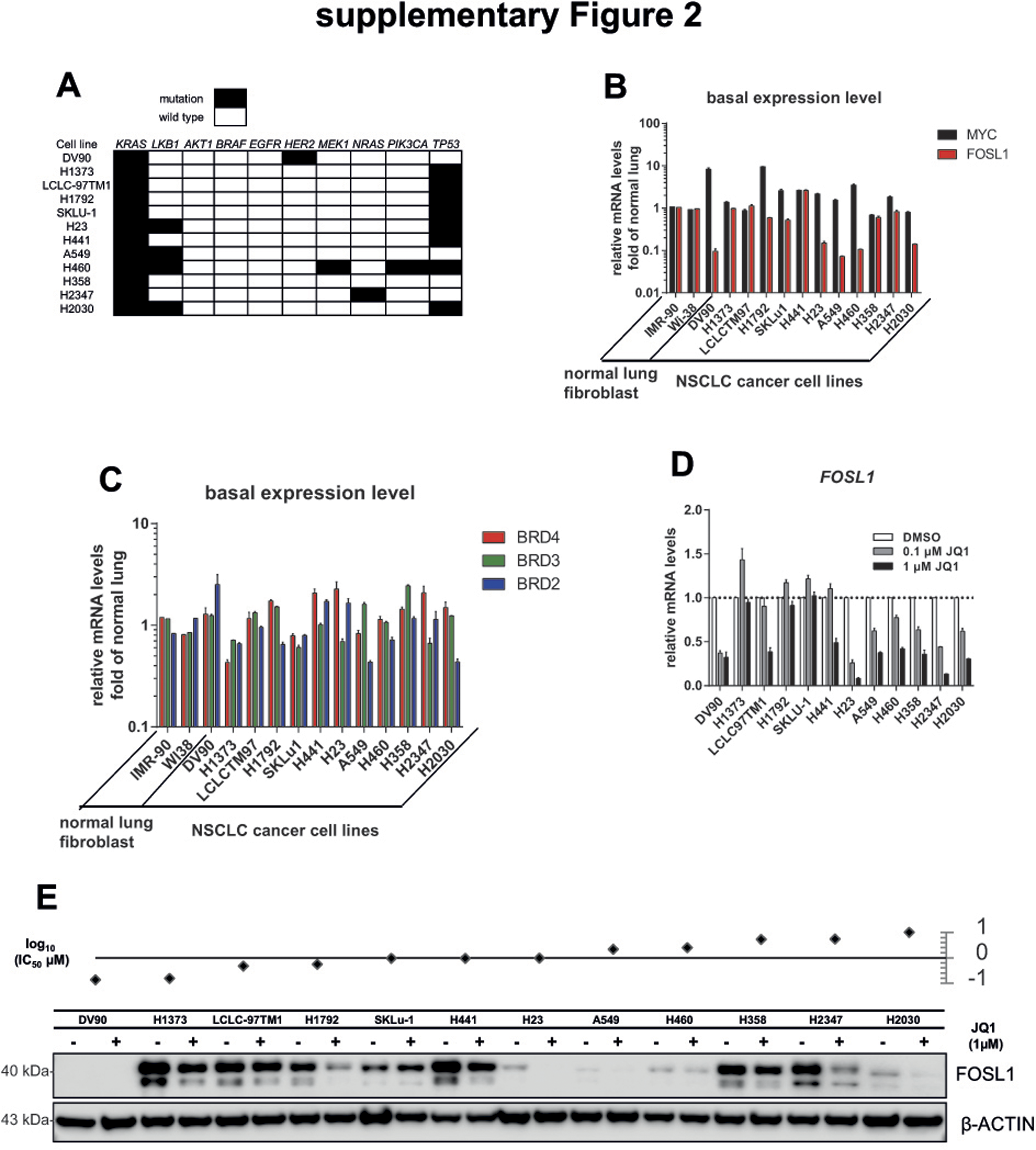

Supplement: Supplementary Figure 2 [file cddis2016271x2.tif]

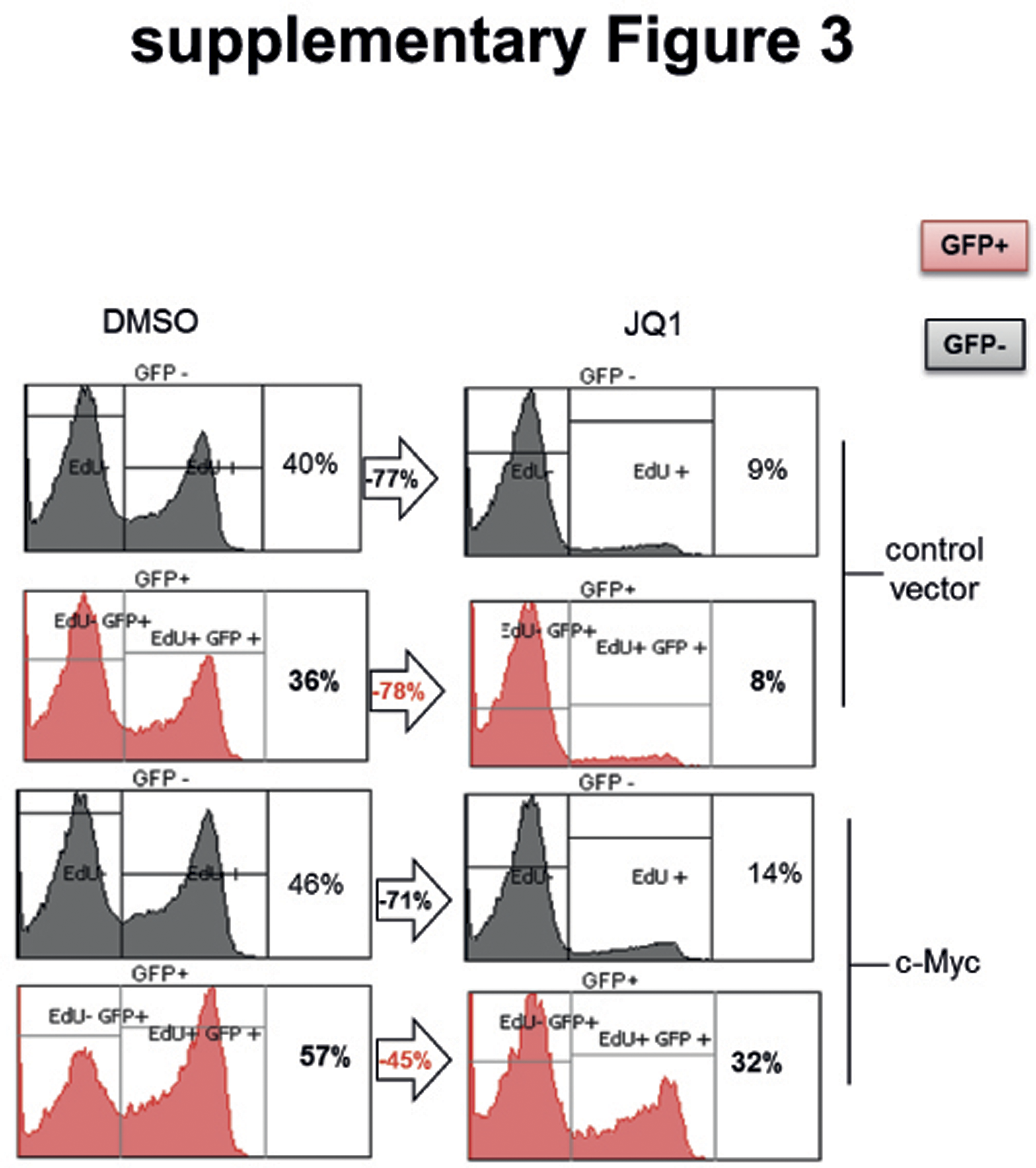

Supplement: Supplementary Figure 3 [file cddis2016271x3.tif]

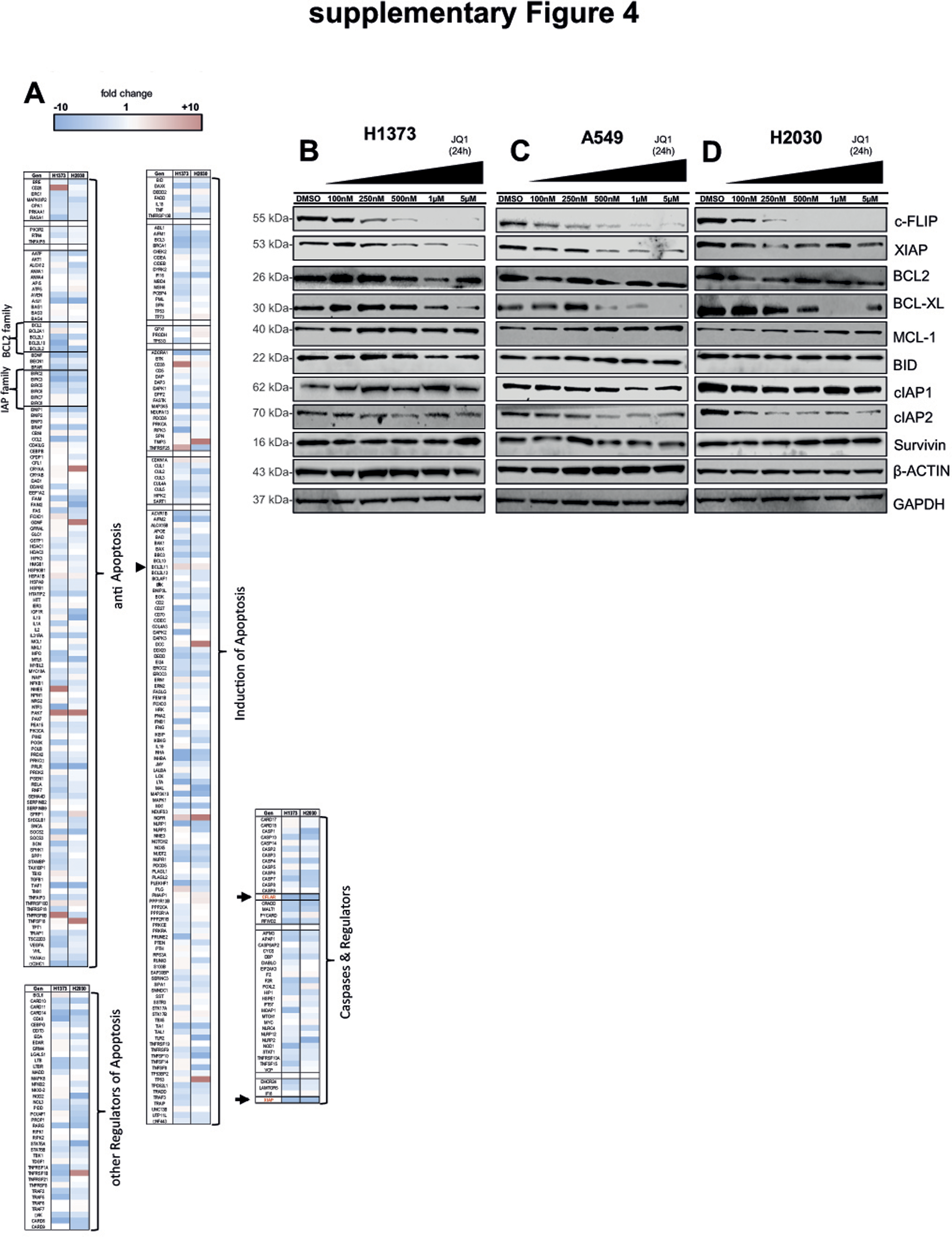

Supplement: Supplementary Figure 4 [file cddis2016271x4.tif]

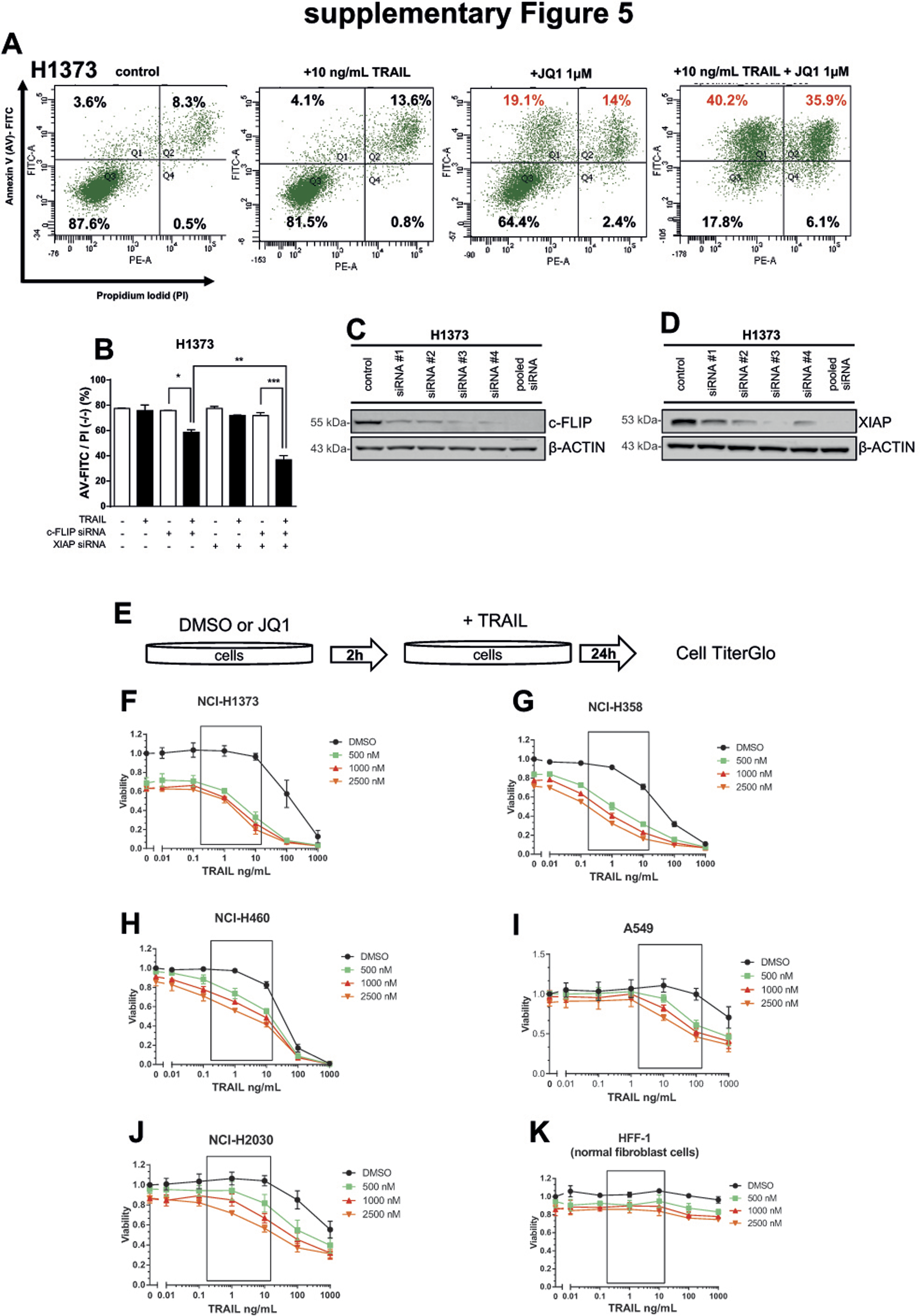

Supplement: Supplementary Figure 5 [file cddis2016271x5.tif]

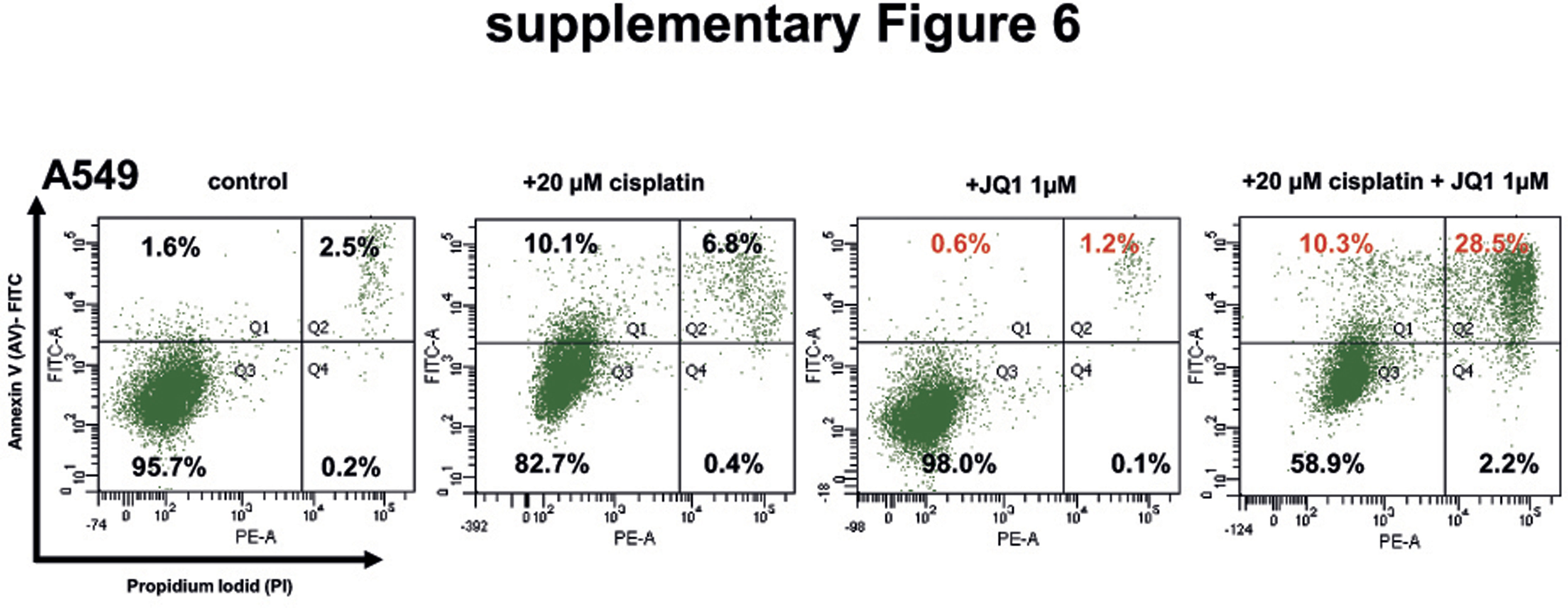

Supplement: Supplementary Figure 6 [file cddis2016271x6.tif]
